# Psychometric responsiveness of the health-related quality of life questionnaire (HeartQoL-P) in the Iranian *post*-*myocardial infarction* patients

**DOI:** 10.1186/s12955-018-1075-8

**Published:** 2019-01-14

**Authors:** F. Ranjandish, H. Mahmoodi, A. Shaghaghi

**Affiliations:** Department of Health Education & Promotion, Faculty of Health, Tabriz University of Medical Sciences, Tabriz, Iran

**Keywords:** Myocardial ischemia, Acute coronary syndrome, Coronary thrombosis, Angina pectoris, Life quality, Persian

## Abstract

**Background:**

Cardiovascular diseases (CVD) as a most frequent and costly NCDs account for about 17.3 million annual deaths worldwide. About 80% of these deaths are taking place in low and middle income countries (LMIC). The survivors may experience severe disabling consequences with extensive impacts on their quality of life. The HeartQoL is a relatively new scale to measure health-related quality of life in CVD patients and was validated for use in other languages. Main aim of the present study was to validate the HeartQoL for Persian speaking populations.

**Design and methods:**

In this cross-sectional study the participants were 557 admitted patients with acute myocardial infarction (AMI) across three specialized hospitals in Tabriz, North West of Iran from Sep 2014 to Feb 2015. Translation back-translation procedures were applied to prepare the Persian version of the HeartQol (HeartQoL-P) and the content validity of the scale was evaluated by an expert panel of 10 academic staff. Construct validity was assessed by exploratory and confirmatory factor analyses. The internal consistency was assessed based on the numeric value of Cronbach’s alpha and sensitivity of the measure according to the ceiling and floor effect’s values.

**Results:**

The two-factor structure of the HeartQoL-P was supported by the confirmatory factor analysis’ outputs and good internal consistency measures (total score α = 0.94) (physical subscale (10 items) α = 0.95) and emotional subscale (4items) α = 0.80)). No ceiling and floor effects were observed for the overall HeartQol-P’s score.

**Conclusion:**

The findings supported the HeartQoL-P usability as a valid instrument in studies on the Iranian or other Persian speaking patients.

## Introduction

Non-communicable diseases (NCDs) are the dominant cause-specific component of death, disability and other socio-economic burden in low and middle income countries (LMIC). [1] Cardiovascular diseases (CVD) as one of the most frequent and costly NCD are the leading cause of death that account for about 17.3 million annual deaths worldwide and this toll is estimated to raise to more than 23.6 million by 2030. [2] More than 80% of all CVD related deaths are taking place in LMIC. [2] The overall global CVD mortality increased almost 41% during 1990–2013 [3] and the burden will continue to grow in the coming decades if an effective intervening measure will not be scheduled.

Acute myocardial infarction is one of the main causes of death in Iran [4, 5] that imposed significant human and non-human costs on the country’s productivity with exacerbated effect on the rural and deprived areas (e.g. adjusted incidence rate of 152.5 per 100,000 population in a relatively deprived province compared to the average countrywide rate of 73.3 per 100,000 population as reported for 2012). [5] Therefore; CVD related mortality or its disabling consequences is one of the greatest challenges for the Iranian National Health System (INHS) and whole country since it was projected that the estimated 847,309 CVD related DALYs (*Disability-Adjusted Life Years)* in the country’s adult population aged ≥30 years old in 2005 to increase more than two fold and reach 1,728,836 by 2025. [6]

Those patients who survive from an acute heart attack may experience severe disabling consequences with extensive impacts on their quality of life for the remaining years. [7] This means that the survivors and their family members’ perceived health, comfort and level of happiness may be diminished in the context of the culture and value systems in which they live and their hopes and dreams ruin due to the disease related hassles in front of reaching their goals, and meeting expectations or standards of living. [8]

Due to usage of a range of condition-specific (MI, heart failure, angina) questionnaires in different studies and considering their inherent inaccuracy and lacking of the required specificity for general usage, cross comparison of the studies’ result on quality of life of the patients with ischemic heart disease (IHD) is absurd. [9] The HeartQoL tool developed by Oldridge N et al. [10] is a relatively new hybrid scale to measure health-related quality of life in patients with IHD and studied on patients across 22 countries and 15 languages. [9] The scale development was based on the three well known condition-specific instruments to measure patients’ quality of life i.e.: (1) MacNew Heart Disease HRQoL (developed for patients with MI), (2) Minnesota Living with Heart Failure, and (3) Seattle Angina Questionnaire [9]. Advantage of this new tool for quality of life measurement in patients with IHD is its easy applicability in clinic and out of clinic settings, relatively short time required for its completion and its efficiency as a core single instrument to be used for other patients with angina, MI and heart failure. [10, 11] The instrument was validated for use in other languages and different types of patients [12, 13] but not validated for use in Persian speaking population. Application of disease-specific instrument to measure quality of life could add sensitivity in differentiating patients’ conditions based upon the imposed burden by an explicit disease. Main aim of the present study therefore, was to validate the HeartQol-P for Iranian post-myocardial infarction patients. .

## Materials and methods

### Study participants

In this cross-sectional study, a convenience sample of discharged patients who had been admitted in the coronary care units with diagnosis of acute myocardial infarction in the previous 4 weeks to 6 months period were recruited from only specialized inpatients CVD wards of three main hospitals in Tabriz the capital city of East Azarbaijan province, North West of Iran. All of the included hospitals are affiliated to the Iranian National Health System (INHS). The targeted patients had been discharged from the hospitals and were approached in their homes.

The patients discharge lists were used to select the stable and eligible patients (aged ≥18 years, diagnosed with AMI, not having any serious cognitive disorder). Thus; 557 patients (410 men and 147 women) after explanation of the study objectives were contacted and invited to participate in the study. The sample size was decided based on the optimum number of participants (20) determined per item in the scale (14 items). Exploratory Factor Analysis (EFA) was performed on a random split half sample of the study data to examine factor structure of the scale’s items and Confirmatory Factor Analysis (CFA) in the holdout sample to validate the identified factor structure.

Data about the general characteristics of the patients who agreed to participate in the study were collected through phone contact, email and face to face questioning. The study aims and objectives were explained orally in face to face meetings (66) or through telephone call (491). Data collection through telephone call or email was applied only for those patients who were not willing to attend the interview place at the hospitals or stated their preference to answer the study questions by telephone or email.

The informed consent form was sent to the patients’ postal address in the printed format upon their request and they were advised to return the completed form in the case of their willingness to participate. Exclusion criteria was unfamiliarity with Persian language (common in limited sub-samples of the country population due to having a different mother language e.g. Turkic, Kurdish, Gilaki, Mazandarani and Balochi) or having a severe disabling condition such as body paralysis which could considerably affect people’s life. Data about socio-demographic properties including age, sex, educational level and also history of background illnesses including type two diabetes and hypertension and also patients’ lifestyle habits i.e. smoking and daily physical activity level (before and after AMI) were collected for all study respondents between September 2014 and February 2015.

### Instrument (HeartQol)

The HeartQol questionnaire is a relatively new tool for examining health-related quality of life amongst patients with IHD [11] and was validated across 22 countries. [9, 12–16] The HeartQoL’s items are listed after an introductory phrase i.e. “*We would like to know how your heart problem has bothered you and how you have been feeling during the last 4 weeks*” and consist 14 questions in the physical and emotional subscales with answer options ranging from 0 to 3 (poor to better health related quality of life). The original scale’s reliability based on the reported Cronbach alpha was ≥0.80. [17]

Forward–backward translation procedures were applied to prepare the Persian version of the HeartQol. [18, 19] The forward translation was carried out by two fluent speakers of Persian language with good knowledge of English. The back translation process was done by two professional translators and a cardiologist checked the final version for its conformity with the original text. Some modifications were made in the Persian translated version after comparing the English back–translated version with the original scale. To assure face and content validities a panel of 10 health professionals were asked to assess and give their comments on the translated instrument. Lucidity of the wording and easy understandability of the scale’s items were also pilot tested on the 30 study participants and minor corrections or amendments were made consequently. To add efficiency of the data collection procedure the pilot phase collected data were included in the main study. [20]

### Data analysis

Mean and standard deviation of the HeartQol-P’s scores in line with absolute and relative frequencies of the baseline variables were calculated. The ceiling and floor effects (CFEs) were appraised by examining the percentage of scores at the boundaries of the score range (e.g. 0 and 100). [21, 22] The CFEs are a matter of concern if more than 15% of respondents achieve the lowest or highest possible score, respectively. Cronbach’s alpha and Interclass Correlation Coefficient (ICC) were computed for evaluating internal consistency and reliability of the scale and values above 0.7 were deemed to be acceptable. [23]

Exploratory Factor Analysis (EFA) was used to examine any underlying (or latent) relationships between the variables. EFA was carried out by Principal Axis Factoring (PAF) extraction method utilizing Varimax Rotation with Kaiser Normalization. The scree plot procedure was used for deciding on the number of factors to be extracted. [24]

KMO (Kaiser-Meyer Olkin) measure of sampling adequacy, Bartlett’s test of sphericity and total explained variance were used for the evaluation of model sufficiency. High values of KMO (more than 0.7) generally indicate that a factor analysis may be suitable for a data set. Bartlett’s test of sphericity speculates the hypothesis that whether a correlation matrix is an identity matrix (i.e. variables are unrelated and therefore unsuitable for structure detection). A *P* value less than 0.05 was considered statistically significant. Factor loading values equal to 0.6 and above were interpreted as representing a good convergent validity or correlation between the items of the identified factors. [25] Confirmatory Factor Analysis (CFA) output was examined to assess construct validity of the adapted instrument. The CFA fit indices and their considered acceptable values were Chi-squared/df < 5.00, Root Mean Square Error of Approximation (RMSEA) < 0.08, Comparative Fit Index (CFI), Goodness of Fit Index (GFI) and Adjusted Goodness of Fit Index (AGFI) > 0.90. [26] The IBM SPSS (version 19) and AMOS (version 18) software was used for data analysis.

## Results

### Content validity

#### Socio-demographic characteristics

All of the invited patients (557 including 410 (73.6%) men and 147 (26.4%) women) agreed to participate and completed the study questionnaire (response rate 100%).The respondents’ age range was from 29 years to 92 years (mean = 62.7 years; SD = 12.3 years). Other characteristics of the study participants were indicated in Table 1. The HeartQoL-P total mean score for all patients was 19.43 (±9.86) and a statically significant difference was observed between two sexes (*p* < 0.001) (males’ mean score = 18.36, SD: 9.92 and females’ mean score = 22.43, SD: 9.05). *No ceiling and floor effects* were observed for the overall HeartQol-P’s score. The content validity ratio (CVR) and content validity index (CVI) represented acceptable ranges i.e. 0.62 and 0.81, respectively. [26]

**Table 1:** The participants’ characteristics in the psychometric study of the health-related quality of life questionnaire (HeartQoL-P) in the Iranian post-myocardial infarction patients (*n* = 557)

Variables	Frequencies (%)
Sex	
Male	410 (73.6)
Female	147 (26.4)
Age range	
< 50	95 (17.0)
50–70	304 (54.6)
> 70	158 (28.4)
Education (*n* = 545)	
Illiterate	241 (44.2)
Primary & high school certificate	205 (37.6)
Diploma & college degree	83 (15.3)
BSc & higher	16 (2.9)
AMI time	
0–3 months	248 (44.5)
4–6 months	309 (55.5)
Smoking	
Before AMI	259 (46.5)
After AMI	70 (12.6)
Adequate physical activities	
Before AMI	
Daily	78 (14.0)
Occasionally	248 (44.6)
Never	230 (41.4)
After AMI	
Daily	78 (14.0)
Occasionally	187 (33.6)
Never	291 (52.2)
Other co-morbidities	
T2 diabetes	183 (32.9)
Hypertension	420 (75.4)

### Reliability

The estimated Cronbach’s alpha for the translated version of the scale was 0.94 which is in the vicinity of acceptable range for internal consistency measure (above 0.7). The reliability coefficients for the two sub scales of the HeartQol-P were also in the acceptable range i.e. the emotional subscale (4 items, α = 0.80) and the physical subscale (10 items, α = 0.95) (Table 2).

**Table 2:** Scale properties of the HeartQol items in the psychometric responsiveness of the health-related quality of life questionnaire (HeartQoL) in the Iranian post-myocardial infarction patients (*n* = 275)

Scale	Number of Items	Mean	SD	Floor-effect (%)	Ceiling-effect (%)	Cronbach’s α
Emotional	4	1.23	0.75	5.0	3.4	0.80
Physical	10	1.44	0.79	1.1	2.5	0.95
Total	14	1.38	0.70	0.2	1.1	0.94

### Construct validity

#### Exploratory factor analysis

**Fig. 1:**
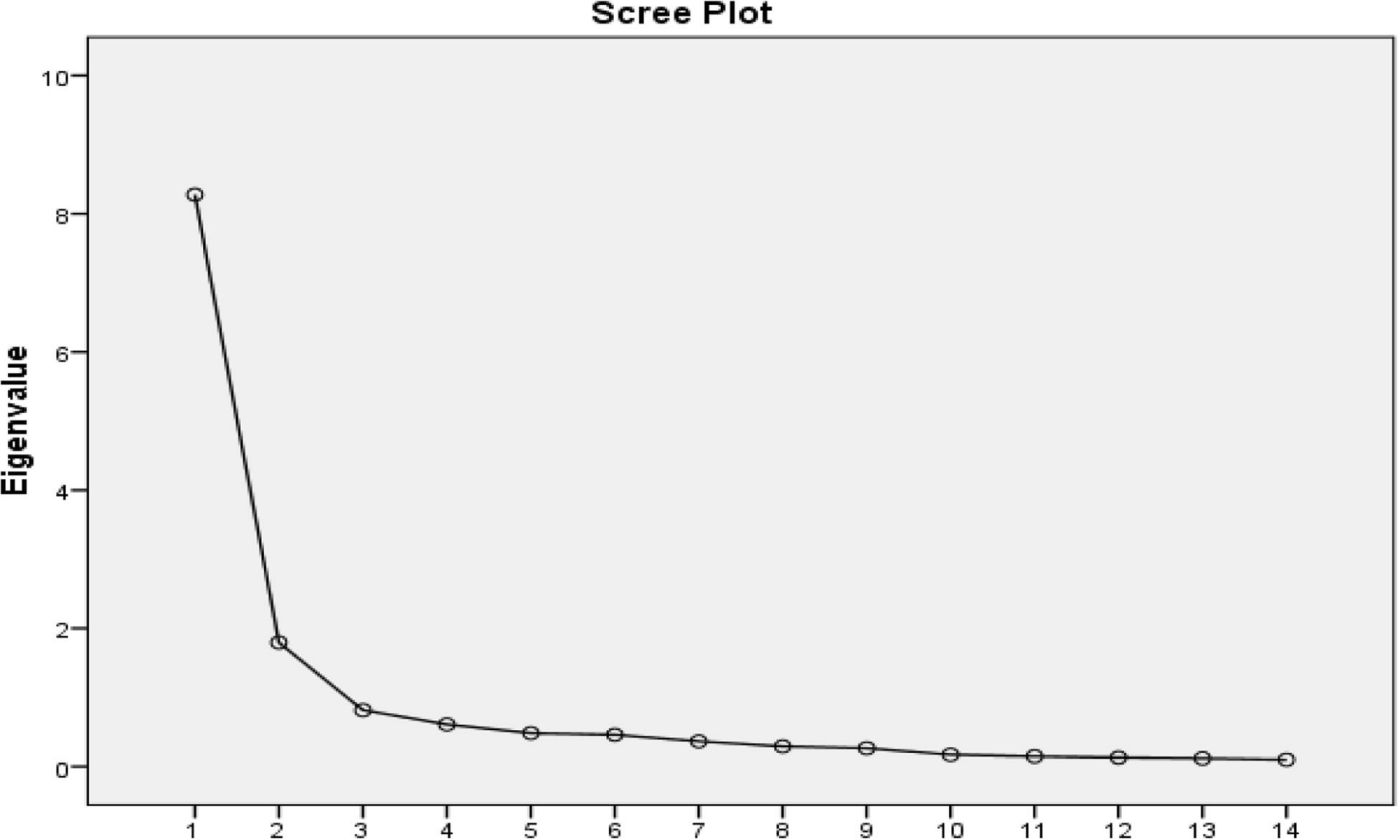
A scree plot of the eigenvalues against all the HeartQol-P’s item numbers

The Exploratory Factor Analysis (EFA) was conducted using data from about half of the study sample (i.e. 275 patients) as recommended when the sample size allows. [27, 28] The output statistics in terms of KMO (Kaiser–Meyer–Olkin) measure of sampling adequacy (0.93) and Bartlett’s test of sphericity (χ (91) = 7345.1, *P* < 0.001) represented EFA usefulness for the data analysis. The total variance explained by the scale’s two factors was 71.93% and the Scree plot supported the unidimensionality of the HeartQol-P questionnaire (Fig. 1).

The factor loadings of the 14 items in the HeartQol-P were presented in Table 3. The identified factors were emotional factor consisting items 9–12 and also physical factor including items 1–8 and 13–14.

**Table 3:** Explanatory Factor Analysis* pattern of matrix loadings for 14 items in the psychometric responsiveness of the translated Health-Related Quality of Life questionnaire (HeartQoL-P) in the Iranian post-myocardial infarction patients (n = 275)

Items	Questions	Factor 1	Factor 2
Q4	Fast walking for more than 100 m	**0.906**	0.160
Q3	Going up stairs or a hill / Going down stairs or a hill without taking a break	**0.897**	0.183
Q13	Exercise intolerance	**0.897**	0.201
Q2	Gardening, vacuuming, carrying groceries	**0.879**	0.224
Q14	Working only at home or yard environment	**0.871**	0.205
Q5	Lifting or moving heavy objects	**0.843**	0.119
Q1	Walking on the ground at home	**0.832**	0.267
Q7	Physical restriction	**0.782**	0.214
Q6	Shortness of breath	**0.720**	0.238
Q8	Tiredness, fatigue, lack of energy	**0.604**	0.465
Q10	Feeling depressed	0.363	**0.797**
Q12	Feeling anxious	−0.052	**0.794**
Q11	Feeling hopeless	0.356	**0.784**
Q9	Feeling under tension or restlessness	0.181	**0.667**

*Explanatory Factor Analysis (EFA) using (Principal Axis Factor) PAF extraction method and varimax rotation with Kaser normalization/bold numbers indicate the items related to the corresponding factor//Factor 1 (physical), Factor2 (emotional)

#### Confirmatory factor analyses (CFA)

**Fig. 2:**
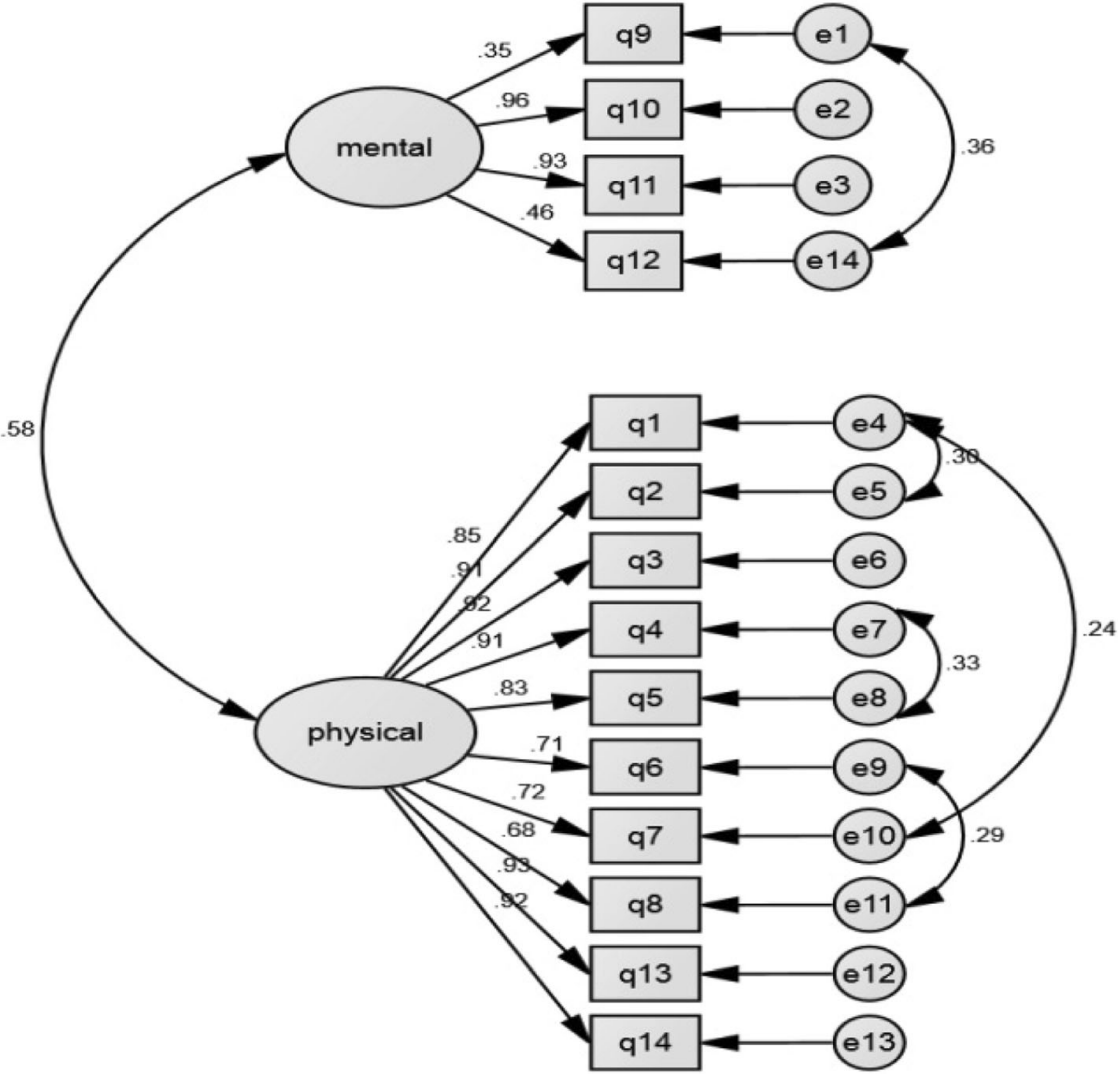
Path diagram of the HeartQol-P’s loading items on the two-factor confirmatory-exploratory model

The results of the CFA for two-factor model indicated a satisfactory fit (X^2^/ df = 2.93 < 5, RMSR (Root Mean Square Residual) = 0.052, RMSEA (90% CI) = 0.084 (0.071; 0.097), CFI = 0.963, PGFI = 0.613, and AGFI = 0.86). The item-to-factors correlations and also the observed correlations among the two factors (Fig. 2) were statistically significant (*P* < 0.05).

## Discussion

The results of this study showed that the translated HeartQol-P questionnaire is a valid and reliable tool for measuring quality of life in patient survived from acute myocardial infarction in the Iranian population.

No floor or ceiling effects (i.e. more than 15% of the study participants produce the maximum or minimum possible scores on a scale) were observed for the total and subscales’ scores of the HeartQoL-P questionnaire which is consistent with the psychometric properties of the main questionnaire. [17] Content validity of the HeartQol-P questionnaire was approved based on both qualitative (i.e. inspection of comments from the expert panel members) and quantitative analysis (i.e. scrutiny of the level of agreement among expert panel members) by examining values of the content validity ratio (CVR) and content validity index (CVI) that represent simplicity, relevancy and clarity of the scale’s items. [10] The translated HeartQol-P questionnaire indicated good internal consistency (the Cronbach alpha for the total score and each subscale was between 0.80 and 0.95). The finding in an agreement with other reported reliability measures in previous studies that ranged from 0.80 to 0.91 [17] and Cronbach alpha (α) ≥ 0.90, test-retest reliability index (ICC) ≥ 0.90 in the study of Kristensen et al. on patients with atrial fibrillation [29], Cronbach α values between 0.88 and 0.92 on the global scale and each subscale in the study of Oldridge et al. ([13] or Cronbach’s alpha > 0.90 and test-retest reliability index of > 0.90 in the study of Zangger et al. [16]

Construct validity of the HeartQol-P was evaluated using EFA and CFA. The results revealed that a two factor model consisting of physical and emotional component was a good fit to the data which is in line with findings of other psychometric studies of the HeartQol. [13, 15–17]

Despite an acceptable representation of the validity and reliability measures, the study findings warrant to be interpreted by caution. Non-random selection of the study participants and their socio-cultural distinct properties could prohibit generelizability of the study findings to other different populations. The age range and gender of the participants might also have effect on their total and subscale’s scores. Time from the AMI event to the study participation date ranged from 4 weeks to 6 months (mean = 3.71 months and SD = 1.63). We believe that different time period after myocardial infarction or clinical severity of the AMI could cause different outcomes and quality of life. Therefore; subgroup analysis based on the different times frames after AMI is recommended for future studies. However; considering main objective of this study (assessment of the psychometric properties of the translated scale) the wide time frame could not be a source of major concern in the interpretation of the study results. Different data collection methods applied in the study i.e. telephone call, face to face interview, email could also pose serious threat to the validity of the study findings. Application of a consistent data collection method is recommended for future studies on the psychometric assessment of the scale. Further research is required therefore; to appraise age adjusted and cross-cultural adaptability of the translated version in different sub-groups of the Iranian population and other Persian speaking countries and also to examine its reliability over a longer time period.

## Conclusion

Perceived quality of life in patient survived from acute myocardial infarction could pose crucial impact on their recovery and sustaining physical and mental wellbeing. Therefore, measurement of the ascertained quality of life in these patients might have a high priority for accurate health care interventions. Findings of this study were indicated that the HeartQol-P questionnaire has the acceptable properties for its use in prediction of quality of life amongst Persian speaking populations. Therefore, its application as a disease sensitive instrument to measure AMI patients’ quality of life in Iran and other Persian speaking countries is recommended however; cross cultural validity of the scale must be investigated in the countries’ sub-groups of populations in future studies.

## Abbreviations

AFGI: Adjusted goodness of fit index
AMI: Acute myocardial infarction
CFA: Confirmatory factor analysis
CVD: Cardiovascular disease
CVI: Content validity index
CVR: Content validity ratio
EFA: Exploratory factor analysis
GFI: Goodness of fit index
HeartQoL: Health-related quality of life questionnaire
ICC: Interclass correlation coefficient
INHS: Iranian national health system
KMO: Kaiser-Meyer Olkin
PAF: Principal axis factoring
RMSEA: Root mean square error of approximation
RSMR: Root-mean-square residuals
SD: Standard deviation
